# The hilly-gully watershed exhibits distinct deep soil moisture characteristics: a comparative study of paired watersheds in the Chinese Loess Plateau

**DOI:** 10.3389/fpls.2025.1668310

**Published:** 2025-09-19

**Authors:** Hongsheng Zhu, Zihan Wang, Jiongchang Zhao, Jiaming Lin, Shuo Qian, Liping Wang, Yang Yu, Marco Cavalli

**Affiliations:** ^1^ School of Soil and Water Conservation, Beijing Forestry University, Beijing, China; ^2^ Jixian National Forest Ecosystem Observation and Research Station, Chinese National Ecosystem Research Network (CNERN), School of Soil and Water Conservation, Beijing Forestry University, Beijing, China; ^3^ College of Biological Sciences, University of Minnesota, Minneapolis, MN, United States; ^4^ National Research Council of Italy, Research Institute for Geo-Hydrological Protection, Padova, Italy

**Keywords:** soil moisture, vegetation restoration, vertical distribution, spatial variability, Loess Plateau

## Abstract

Deep soil moisture constitutes a critical component of hydrological processes in the Loess Plateau, playing an essential role in sustaining vegetation growth, maintaining ecosystem stability, and serving as an important indicator of regional water resource carrying capacity. However, the mechanisms by which long-term vegetation restoration influences deep soil moisture remain insufficiently understood. In this study, we selected two typical paired small watersheds–an Artificial forest watershed and a Farmland watershed-located in the Caijiachuan watershed in the hilly-gully region of the Loess Plateau in western Shanxi Province, China. Based on *in situ* measurements of soil moisture (0–500 cm) during the 2024 growing season (May-October), the vertical distribution and spatial variability of soil moisture have been systematically analyzed, and the impacts of long-term vegetation restoration on deep soil moisture content have been assessed. The results revealed significant differences between the paired watersheds. The average soil moisture content in the Farmland watershed (0.096 g/g) was significantly higher than in the Artificial forest watershed (0.070 g/g), indicating that artificially introduced vegetation has substantially reduced deep soil moisture reserves. Land use has pronouncedly influenced deep soil moisture, with farmland and native grassland exhibiting the highest moisture retention capacity, while vegetation restoration sites showed the lowest levels. Deep-rooted plantations in the Artificial forest watershed markedly intensified soil moisture deficits in the 200–500 cm layers, whereas the Farmland watershed exhibited comparatively moderate deficits. Moreover, soil moisture spatial heterogeneity was significantly greater in the Farmland watershed, while long-term vegetation restoration promoted a more homogeneous distribution of deep soil moisture. Overall, large-scale restoration dominated by deep-rooted species exerted substantial impacts on deep soil moisture dynamics. These findings provide a scientific basis for vegetation restoration planning and watershed management in the Loess Plateau region.

## 1 Introduction

Soil moisture constitutes a vital element of the terrestrial system, serving an integrative function in surface processes, particularly within water-limited regions. In these areas, the availability of soil moisture is regarded as the principal determinant of the land productivity and sustainability of watershed management (Gao et al., 2014). Soil moisture demonstrates significant heterogeneity in both spatial and temporal dimensions, even within small watersheds (Dymond et al., 2021; Wang et al., 2019). The characterization of soil moisture variations across diverse spatial and temporal scales is crucial for theoretical understanding and practical applications related to surface runoff and erosion, agriculture management, and ecological restoration (Chatterjee et al., 2022; de Queiroz et al., 2020; Deng et al., 2016).

Soil moisture presents different characteristics in time and space. Numerous studies indicate that soil moisture is affected by a range of environmental factors, including land use, vegetation types, topographical conditions, and soil properties (Fathololoumi et al., 2021; Xu et al., 2021; Yu et al., 2018, 2019). For example, at the hillslope scale, topographic conditions such as slope position and aspect significantly affect soil moisture distribution, with shady slopes and lower positions generally exhibit higher moisture levels due to reduced evaporation and runoff accumulation (Dong et al., 2018); at the watershed scale, land use type, soil properties, and topographical conditions are dominant factors shaping soil moisture variability, where grassland and farmland exhibit differing retention capacities due to root structure and infiltration rates (Huang et al., 2016; Shi et al., 2012); at the regional scale, climatic factors such as precipitation and evapotranspiration predominantly govern soil moisture dynamics, directly influencing moisture content, spatial variability, seasonal fluctuations, and interannual variability (Wang et al., 2012).

Although soil properties and topography tend to remain constant in the short term, land use and climate factors represent the principal dynamic variables (Montenegro and Ragab, 2012; Sharma et al., 2022). On one hand, alterations in land use can significantly disrupt the surface water balance, influencing the allocation of precipitation among evapotranspiration, runoff, and groundwater flow. This, in turn, plays a critical role in determining the distribution of soil moisture within ecosystems. On the other hand, deep soil moisture is less sensitive to short-term climatic fluctuations and land surface changes, but is more closely linked to long-term patterns of infiltration and groundwater dynamics. Studies have shown that deep-layer moisture is predominantly governed by soil texture, soil thickness, topographic wetness index, and antecedent precipitation, with vegetation root depth also contributing significantly to the vertical redistribution of water (Li et al., 2021; Yu et al., 2019). Unlike surface moisture, which responds rapidly to rainfall and evapotranspiration, deep soil moisture content reflects integrated hydrological processes over longer timescales and thus serves as a more stable indicator of ecohydrological conditions (Wang et al., 2024a, 2024).

The Loess Plateau is an environmentally sensitive area, soil loss and water erosion are the main environmental problems in this area. Since 1999, the “Grain to Green Program” has promoted introduced vegetation to combat soil erosion, vegetation restoration proving to be the most effective measure in ecological management (Bai et al., 2024; Wang et al., 2021; Yu et al., 2020). Despite significant progress in ecological engineering, large-scale revegetation has led to excessive water consumption, resulting in dried soil layers and stunted old trees, thus weakening the expected effect of afforestation in reducing water loss and improving the local environment (Mei and Ma, 2022; Yao et al., 2016; Yu et al., 2024). Deep soil moisture is difficult to replenish. A decline in soil moisture has many other consequences for local water resources. For instance, Wang et al. (2024) found that in China’s monsoon loess critical zone, intensive land-use conversions since 1999 have been the predominant catalyst of deep soil moisture decline, exceeding the effects of climate change and threatening soil moisture security across the Loess Plateau. Cheng et al. (2020) reported that in the semi-arid regions of northern China, vegetation restoration has significantly improved precipitation utilization. However, it has inhibited the recharge of deep soil moisture. Jia and Shao, 2014 emphasized that vegetation type significantly affects the dynamics of deep soil moisture. In addition to the increasing potential evapotranspiration, plant water consumption has substantially degraded soil moisture conditions in the experimental plots.

Soil moisture monitoring at the watershed scale is a crucial component in understanding the interactions between water and vegetation. Recent research on small watersheds has predominantly concentrated on slopes or individual land-use types. However, there is a paucity of studies examining the impact of vegetation restoration on deep soil moisture at the watershed scale. The implementation of paired watershed monitoring is a primary method for elucidating hydrological processes; nevertheless, few studies have systematically examined deep water dynamics paired with watersheds. In this study, a paired-site methodology has been employed to assess the variation and magnitude of soil moisture resulting from long-term vegetation restoration in the hilly-gully region of the Loess Plateau, China. The main research purposes of this study were (i) to compare the differences of soil moisture variation across layers between paired watersheds; (ii) to explore the relationship between land uses and soil moisture variability, and (iii) to examine the effects of long-term vegetation restoration on deep soil moisture distribution.

## 2 Materials and methods

### 2.1 Study area

**Figure 1:**
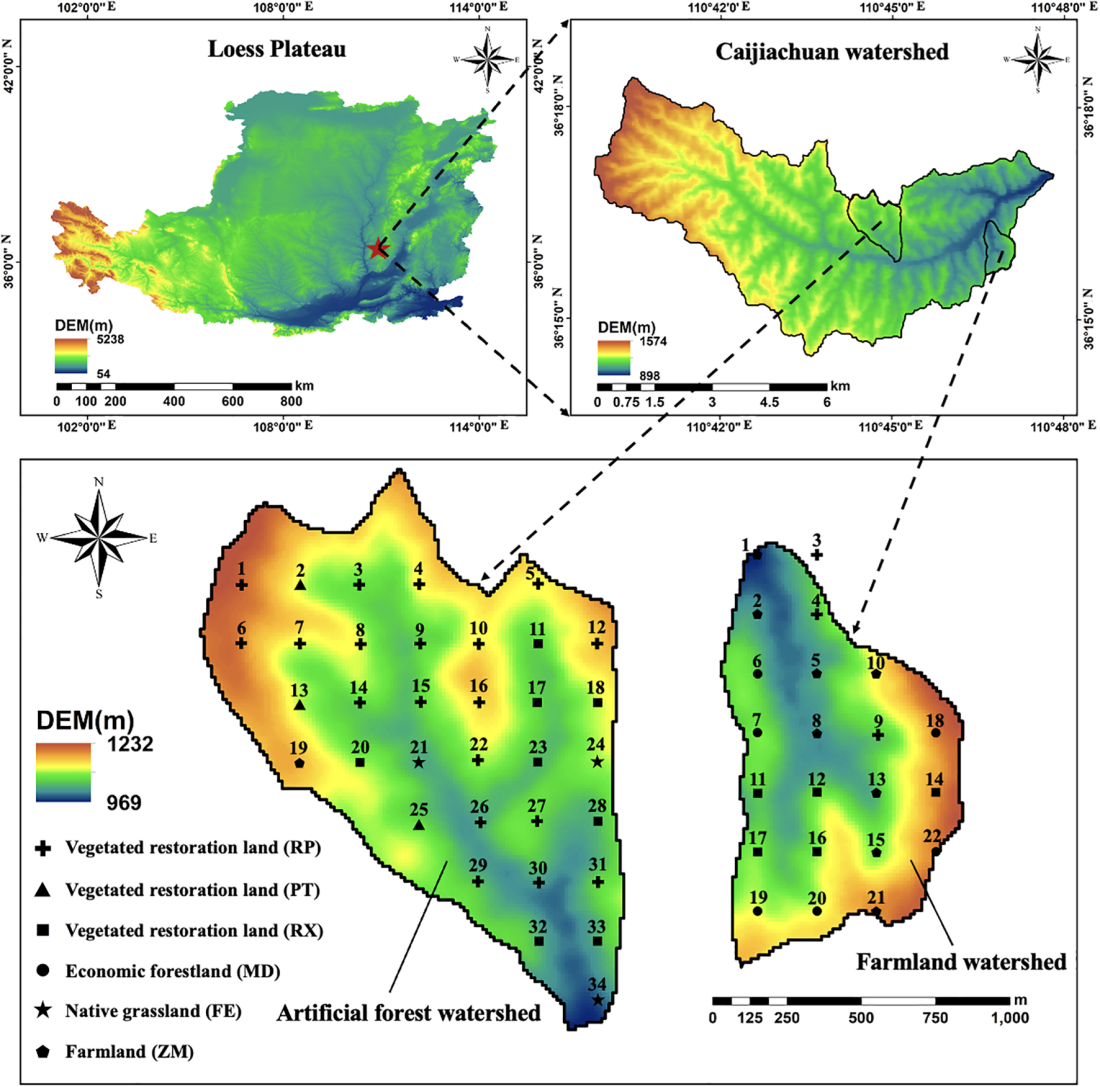
Location of the study area and experimental sites in two watersheds.

The study area is located in the Caijiachuan watershed in the Loess Plateau (**Figure 1**) in the western part of Shanxi Province, China, with geographical coordinates ranging from 110°39′45″ to 110°47′45″E and 36°14′27″ to 36°18′23″N. Since afforestation began in 1990, the forest coverage rate in the watershed has exceeded 80%, resulting in the establishment of approximately 1,000 hectares of soil and water conservation forest. The tree layer is dominated by *Robinia pseudoacacia* (RP) and *Pinus tabuliformis* (PT), the shrub layer by *Rosa xanthina* (RX), and the herbaceous layer by *Festuca elata* (FE). The Artificial forest watershed and the Farmland watershed are two paired watersheds affiliated with the Caijiachuan watershed. Prior to vegetation restoration, the Artificial forest watershed was mainly farmland and shared similar land-use characteristics with the current Farmland watershed, which is geographically adjacent and thus selected as a reference (paired) watershed. Both watersheds are located in a typical loess residual plateau gully region, characterized by brown soils derived from loess parent material. The area experiences a warm-temperate continental climate, with rainfall primarily concentrated between June and September. The annual sunshine duration is approximately 2,563 hours, the average annual precipitation is 579 mm, and the average potential evapotranspiration is 1,723.9 mm. Rainfall patterns exhibit a uniform spatial distribution across the two watersheds. The Artificial forest watershed is situated in the central part of the Caijiachuan watershed, with an elevation ranging from 969 to 1,232 m and a total area of 1.52 km². It features high terrain in the northwest and low terrain in the southeast, with a general north-south orientation. Land use is dominated by vegetation restoration species (RP, PT, RX). In contrast, the Farmland watershed lies in the eastern part of the Caijiachuan watershed, with an elevation ranging from 915 to 1,110 m and an area of 0.76 km². Its terrain is high in the southeast and low in the northwest, also aligned in a north-south direction. The dominant land-use types are rain-fed farmland planted with *Zea mays* (ZM) and economic forest with *Malus domestica* (MD). The introduced vegetation types in the Caijiachuan watershed include RP, PT, and RX.

### 2.2 Field monitoring and experimental design

In the Artificial forest watershed and the Farmland watershed, there are different land use types. This study focused on 4 different land use types with different dominant plant species on them, which are native grassland (*Festuca elata*, FE), vegetated restoration land (*Robinia pseudoacacia*, RP; *Pinus tabuliformis*, PT; *Rosa xanthina*, RX), economic forestland (*Malus domestica*, MD), and farmland (*Zea mays*, ZM). In the Artificial Forest watershed, there were three land use types: native grassland, vegetated restoration land, and farmland, and in the Farmland watershed, there are also three land use types, which are vegetated restoration land, farmland, and economic forestland. Considering that the soil properties of the Artificial forest watershed and the Farmland watershed are generally consistent, this study, based on topographic characteristics and vegetation distribution, has established 34 experimental sites in the Artificial forest watershed: 30 of them were located within vegetated restoration land (including 19 in RP, 3 in PT, and 8 in RX), 3 of them were in native grassland (FE), and 1 of them were in farmland (ZM) (**Table 1**), and 22 experimental sites in the Farmland watershed: 8 of them were in vegetated restoration land (including 3 in RP and 5 in RX), 6 of them were in economic forestland (MD), and another 8 of them were in farmland (ZM) (**Table 2**), in August 2024 using a grid-based sampling design. At each experimental site, soil samples were collected with a soil auger to measure the gravimetric soil moisture (g/g) within the 0–500 cm soil profile. Given the pronounced topographic variability and the complex distribution of vegetation types across the Loess Plateau, the grid-based layout was adopted to enhance the spatial representativeness of the experimental sites, facilitate comparisons of soil moisture status among different land use types, and provide a robust foundation for subsequent modeling of spatial variability in deep soil moisture. During the field surveys, slope gradient, slope aspect, vegetation type, and vegetation cover were specifically documented at each experimental site. In addition, a handheld GPS device was used to measure and record the latitude, longitude, and elevation of each site.

**Table 1:** Topographic and vegetation characteristics of monitoring plots in the Artificial forest watershed.

Land use	Vegetation type	Slope position	Elevation (m)	Slope gradient (°)	Slope aspect (°)	Sample no.
Vegetated restoration land	RP	Upper Slope	1210.13	20.24	102.53	1
		Lower Slope	1072.56	10.27	186.34	3
		Middle Slope	1126.41	25.10	253.89	4
		Middle Slope	1126.09	25.10	253.89	5
		Upper Slope	1128.54	16.61	193.57	6
		Middle Slope	1197.23	27.75	81.25	7
		Lower Slope	1121.94	36.03	161.57	8
		Lower Slope	1113.4	27.54	85.60	9
		Middle Slope	1063.21	14.62	302.47	10
		Middle Slope	1066.33	14.37	218.66	12
		Lower Slope	1107.65	29.10	72.22	14
		Lower Slope	1064.76	25.57	206.03	15
		Upper Slope	1067.77	29.50	261.87	16
		Middle Slope	1035.26	10.77	86.99	22
		Lower Slope	1072.49	28.88	22.38	26
		Middle Slope	1034.67	21.41	289.36	27
		Lower Slope	1049.71	23.41	263.37	29
		Lower Slope	1054.66	22.49	37.15	30
		Middle Slope	1020.12	27.38	169.99	31
	PT	Middle Slope	1119.27	29.39	96.12	2
		Middle Slope	1152.38	21.22	281.89	13
		Middle Slope	1097.54	16.70	216.87	25
	RX	Lower Slope	1135.44	19.00	334.18	11
		Lower Slope	1148.94	16.70	216.87	17
		Lower Slope	1063.34	20.16	60.64	18
		Middle Slope	1167.21	17.87	97.13	20
		Lower Slope	1098.18	16.56	222.27	23
		Middle Slope	1069.03	16.40	99.78	28
		Middle Slope	1068.11	18.99	305.54	32
		Middle Slope	1037.76	29.63	79.88	33
Native grassland	FE	Lower Slope	1094.72	31.53	19.03	21
		Middle Slope	1047.9	9.66	139.76	24
		Lower Slope	994.43	29.62	230.71	34
Farmland	ZM	Upper Slope	1110.06	23.31	248.20	19

FE, native grassland (*Festuca elata*); RP, PT, and RX, vegetated restoration land (*Robinia pseudoacacia*, *Pinus tabuliformis*, and *Rosa xanthina*, respectively); ZM, farmland (*Zea mays*).

**Table 2:** Topographic and vegetation characteristics of monitoring plots in the Farmland watershed.

Land use	Vegetation type	Slope position	Elevation (m)	Slope gradient (°)	Slope aspect (°)	Sample no.
Farmland	ZM	Lower Slope	917.13	7.97	278.97	1
		Lower Slope	955.02	14.20	71.57	2
		Lower Slope	961.23	17.87	299.75	5
		Lower Slope	950.96	5.11	206.57	8
		Middle Slope	1033.14	24.36	276.34	10
		Middle Slope	969.19	23.94	262.24	13
		Upper Slope	1006.23	4.85	45.00	15
		Upper Slope	1082.56	5.88	330.95	21
Economic forestland	MD	Middle Slope	984.77	30.74	70.35	6
		Middle Slope	1002.1	11.31	306.87	7
		Upper Slope	1081.54	30.22	254.06	18
		Upper Slope	1029.43	19.69	26.57	19
		Upper Slope	1030.55	19.53	338.50	20
		Upper Slope	1083.78	8.66	246.80	22
Vegetated restoration land	RP	Middle Slope	993.36	5.42	288.43	3
		Middle Slope	975.31	24.19	253.18	4
		Middle Slope	983.09	20.24	192.53	9
	RX	Lower Slope	982.83	13.52	196.93	11
		Middle Slope	995.27	28.29	318.01	12
		Upper Slope	1075.48	16.61	283.57	14
		Middle Slope	1006.59	33.10	302.47	16
		Middle Slope	1004.05	13.61	51.71	17

ZM, farmland (*Zea mays*); MD, economic forestland (*Malus domestica*); RP and RX, vegetated restoration land (*Robinia pseudoacacia* and *Rosa xanthina*, respectively).

### 2.3 Data collection

In the growing season of 2024 (May to October), soil moisture content in the 0–500 cm layer was measured at each experimental site. Three sampling profiles were randomly selected per site to obtain the average soil moisture content. Soil samples were collected with a 5 cm diameter auger at 10 cm intervals for the 0–100 cm layer and 20 cm intervals for the 100–500 cm layer, each profile yielded 30 samples, generating a total collection of 5040 samples across all study sites. According to previous studies conducted in the Loess Plateau (Yu et al., 2019), the annual precipitation infiltration depth generally does not exceed 200 cm, and soil moisture below this depth exhibits relatively small fluctuations and remains comparatively stable. Therefore, in this study, the 0–200 cm layer was defined as shallow soil, while the 200–500 cm layer was defined as deep soil. Samples were immediately sealed in aluminum cylinders after collection and weighed. They were then dried in an oven at 105°C until a constant weight was achieved. Soil moisture content (g/g) was calculated as the ratio of the mass lost during oven drying to the constant dry weight.

To analyze the spatial distribution of soil moisture, the Inverse Distance Weighting (IDW) method was used to interpolate soil moisture content and generate spatial distribution maps at different depths for both watersheds. ArcGIS software was employed for IDW interpolation and mapping, and its spatial analysis tools were used to perform basic statistical analyses based on the interpolated data.

### 2.4 Statistical analysis

The soil moisture content and profile distribution in each watershed was calculated by taking the average of all experimental sites in each soil layer. The depth-averaged gravimetric soil moisture content $\theta_{ij}$ (g/g) at each experimental site was calculated by Equation 1.


(1) $$\theta ij=\frac{1}{i}\sum_{i=1}^{i}\theta i$$

where *I* is the number of measured strata at site *j* and $\theta_{ij}$ is the soil moisture content of stratum *i* calculated from three randomly sampled profiles. In the following analysis, gravimetric soil moisture content is calculated on a per-100-centimeter basis, with *I* = 5.

The depth-averaged gravimetric soil moisture content $\theta_{m}$ (g/g) for each land use type was calculated by Equation 2.


(2) $$\theta m=\frac{1}{k}\sum_{k=1}^{k}\theta ij$$

where *k* is the number of experimental sites for each land use type.

The depth-averaged volumetric soil moisture content $\theta_{v}\;$ (%) for each land use type was calculated by Equation 3.


(3) θv=θmi×ρbi

where $\rho b_{i}$ is the average soil bulk density of layer *i* for each land use type.

The soil moisture content depletion degree (SMCD) (%) was calculated by Equation 4.


(4) $$SMCD=\frac{1}{k}\sum_{k=1}^{k}\frac{\theta vl-\theta vc}{\theta vl}$$

where $\theta_{vl}$ represents the volumetric soil moisture content of the layer *i* of farmland (*Zea mays*, ZM), and $\theta_{vc}$ represents the volumetric soil moisture content of the layer *i* under different vegetation types.

This study utilized the Inverse Distance Weighting (IDW) method within ArcGIS 10.3 to interpolate and analyze the spatial distribution of soil moisture in the Artificial Forest Watershed and the Farmland Watershed (Gao and Yang, 2023). Spatial distribution maps of soil moisture were generated using this method. IDW estimates the values of unsampled locations through a linear weighting of known sample sites, where the weights are inversely proportional to the distance between the sample and the target sites. The IDW method was calculated by Equation 5.


(5) $$\hat{\text{Z}}(\text{u})=\frac{\sum_{i=1}^{n}\frac{Zi}{d{\left(u,ui\right)}^{P}}}{\sum_{i=1}^{n}\frac{1}{d{\left(u,ui\right)}^{P}}}$$

where $\hat{\text{Z}}(\text{u})$ is the estimated value at the unknown site *u*; *Zi* is the observed value at the known site *u_i_*; *d*(*u,u_i_*) represents the distance between the unknown site *u* and the known site *u_i_*; and *P* is the power parameter that controls the influence of distance on the weighting (Pal and Maity, 2021).

Basic descriptive statistics, including the mean, standard deviation (SD), and coefficient of variation (CV), were calculated for each measurement. Prior to conducting the paired t-test and one-way analysis of variance (ANOVA), the Shapiro–Wilk test was applied to assess the normality of the data. Variables that did not meet the normality assumption were log-transformed before statistical analysis. A paired t-test was utilized to examine the differences in soil moisture across soil layers between the two watersheds. To assess the impact of various land use types on the overall variability of soil moisture, a one-way analysis of variance (ANOVA) was conducted. *Post hoc* multiple comparisons were executed using the Least Significant Difference (LSD) method. Microsoft Excel was employed for preliminary data organization, whereas SPSS and Origin were used for advanced statistical analysis and data visualization.

## 3 Results

### 3.1 Vertical soil moisture variation

**Figure 2:**
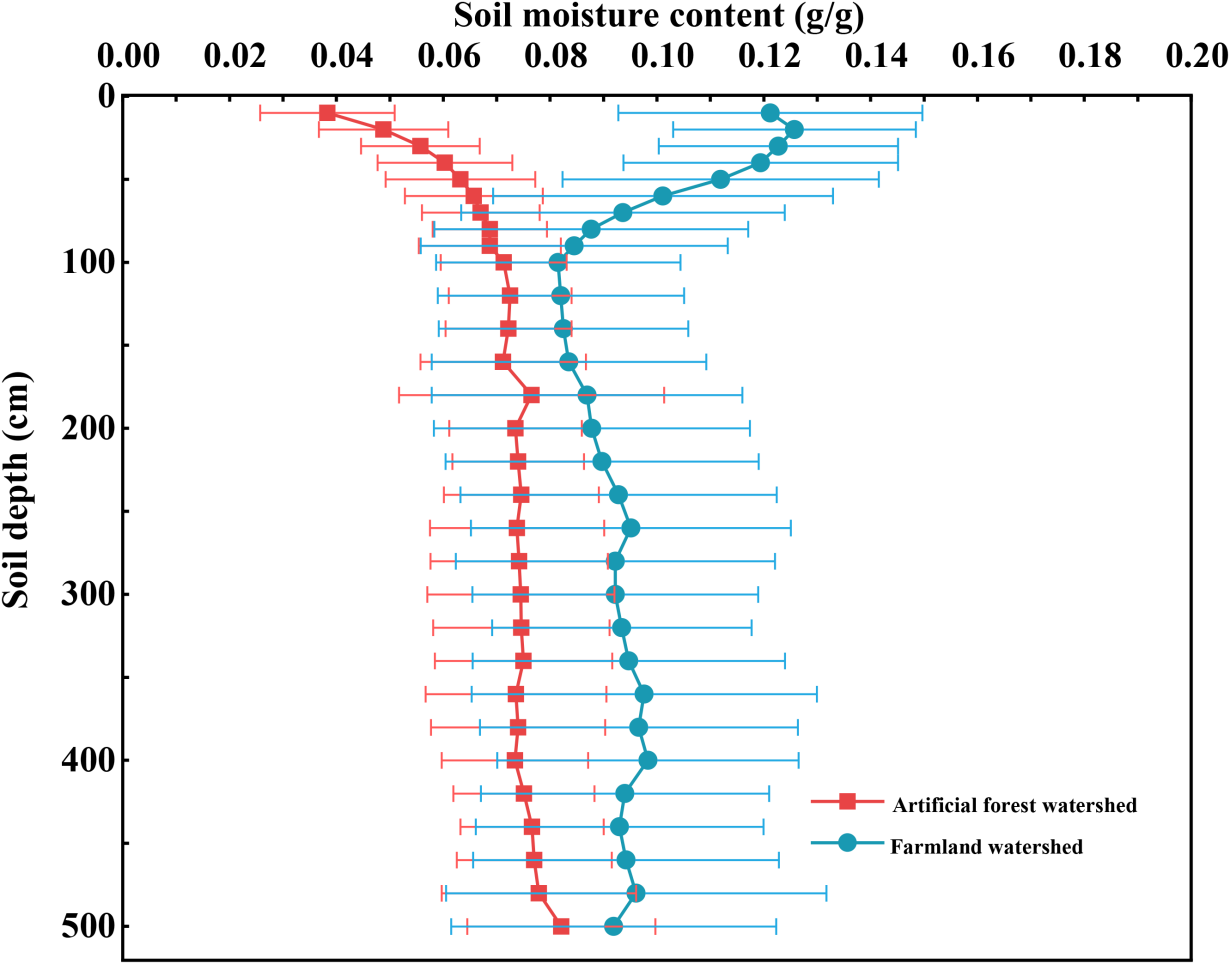
Profile distribution of the mean soil moisture content in two watersheds, the error bars indicate standard deviation.

As shown in **Figure 2**, a significant difference has occurred in soil moisture content between the two watersheds (*p* < 0.05). Soil moisture in the Artificial forest watershed ranged from 0.038 to 0.082 g/g, whereas in the Farmland watershed, it ranged from 0.082 to 0.130 g/g, the overall soil moisture content in the Farmland watershed is higher than that in the Artificial forest watershed. In terms of vertical distribution, the average soil moisture content in the Artificial forest watershed gradually increased with soil depth before stabilizing, whereas in the Farmland watershed, it originally decreased, then increased, and eventually stabilized. The standard deviation of soil moisture in the Farmland watershed was greater than in the Artificial forest watershed.

**Figure 3:**
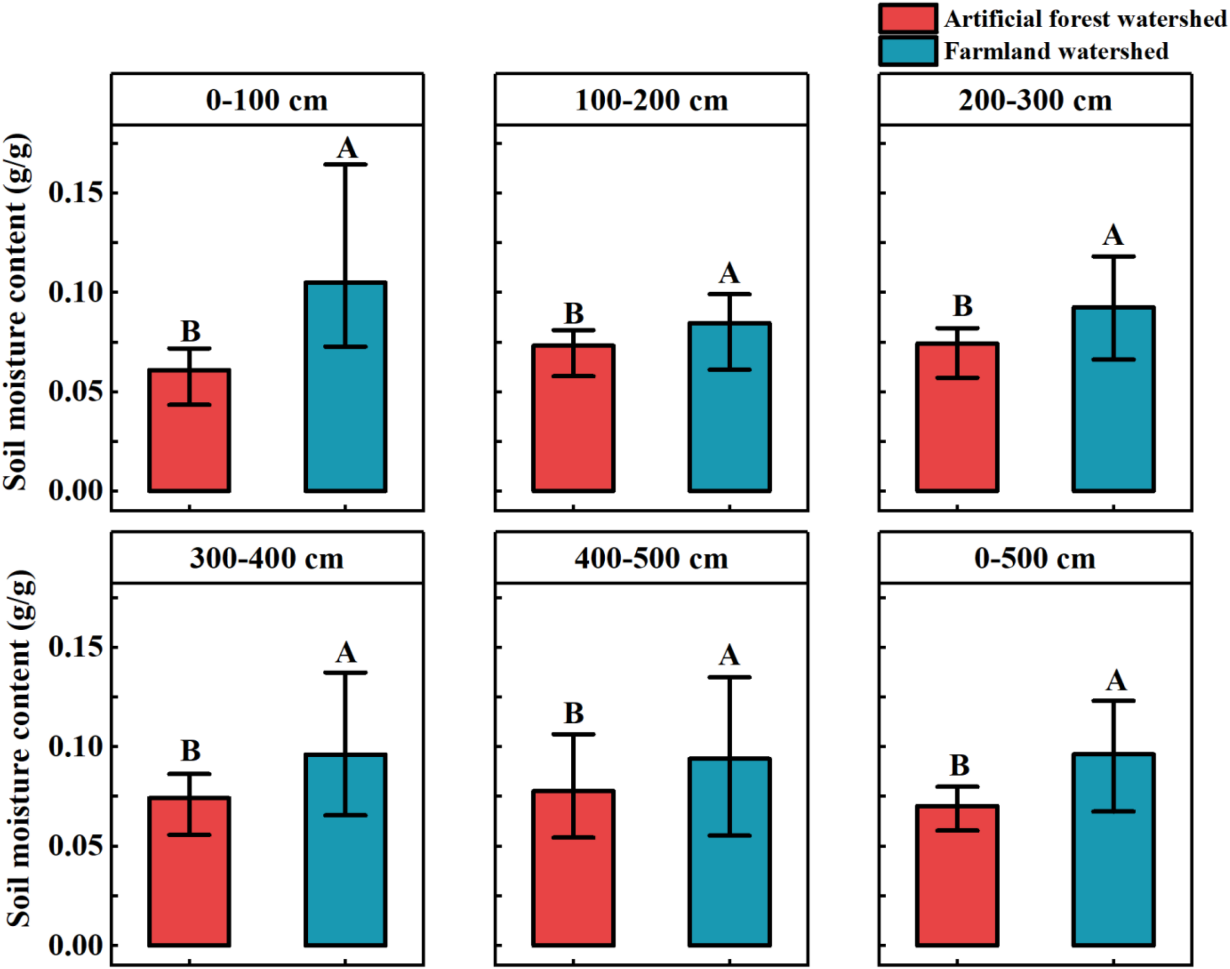
Comparison of soil moisture content of different soil layers in two watersheds, error bars indicate standard deviation, different uppercase letters above the bars indicate significant differences in soil moisture content between the two watersheds.

As shown in **Figure 3**, there was a significant difference (*p* < 0.05) in the average soil moisture content across different soil layers between the Artificial forest watershed and the Farmland watershed. Specifically, in the 0–100 cm soil layer, the average soil moisture content in the Farmland watershed was 0.105 g/g, which was 72.13% higher than in the Artificial forest watershed (0.061 g/g). In the 100–200 cm soil layer, soil moisture in the Farmland watershed was 0.085 g/g, 16.44% higher than in the Artificial forest watershed (0.073 g/g). In the 200–300 cm soil layer, the Farmland watershed recorded 0.092 g/g, 24.32% higher than the Artificial forest watershed (0.074 g/g). In the 300–400 cm soil layer, the moisture content in the Farmland watershed was 0.096 g/g, 29.73% higher than in the Artificial forest watershed (0.074 g/g). In the 400–500 cm soil layer, it was 0.094 g/g in the Farmland watershed, 20.51% higher than in the Artificial forest watershed (0.078 g/g). Overall, the average soil moisture content in the 0–500 cm layer in the Farmland watershed was 0.096 g/g, representing a 37.14% increase compared to the Artificial forest watershed (0.070 g/g).

### 3.2 Soil moisture of different land use types in paired watersheds

**Figure 4:**
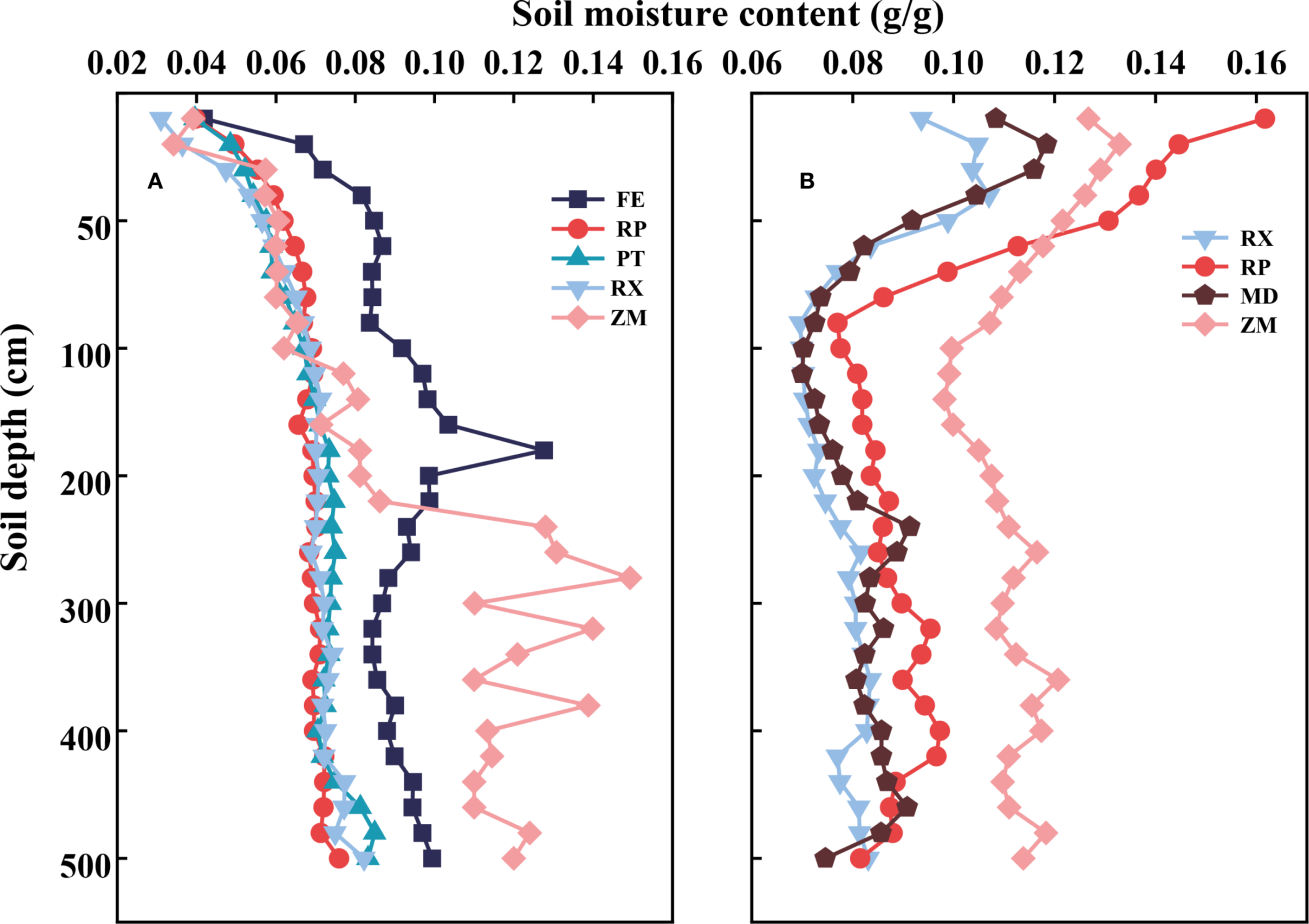
Soil moisture in different land use types in two watersheds **(A)** Artificial forest watershed and **(B)** Farmland watershed. FE, native grassland (*Festuca elata*); RP, PT, and RX, vegetated restoration land (*Robinia pseudoacacia*, *Pinus tabuliformis*, and *Rosa xanthina*, respectively); ZM, farmland (*Zea mays*); MD, economic forestland (*Malus domestica*).

In the two watersheds, soil moisture content was highest in farmland (ZM), followed by native grassland (FE), and lower in the vegetated restoration land (RP, PT, RX), and economic forestland (MD) (**Figure 4**; **Table 3**). After the LSD test (**Table 3**), the differences in soil moisture between different vegetation types in the same watershed were statistically significant (*p* < 0.05).

**Table 3:** Soil moisture (g/g) of 0–500 cm soil layers in different land use types.

Land use	Soil depth (cm)	Native grassland	Vegetated restoration land			Farmland	Economic forestland
		FE	RP	PT	RX	ZM	MD
Artificial forest watershed	0–100	0.078 ± 0.016a	0.060 ± 0.010b	0.056 ± 0.009b	0.055 ± 0.014b	0.056 ± 0.011b	
	100–200	0.105 ± 0.018a	0.068 ± 0.046b	0.071 ± 0.003b	0.071 ± 0.003b	0.078 ± 0.004b	
	200–300	0.092 ± 0.005a	0.070 ± 0.005b	0.073 ± 0.001b	0.079 ± 0.006b	0.121 ± 0.024a	
	300–400	0.087 ± 0.005a	0.070 ± 0.005b	0.072 ± 0.003b	0.077 ± 0.005b	0.125 ± 0.014a	
	400–500	0.095 ± 0.006a	0.073 ± 0.004b	0.079 ± 0.006b	0.071 ± 0.013b	0.116 ± 0.006a	
	0–500	0.091 ± 0.021a	0.068 ± 0.010b	0.070 ± 0.012b	0.071 ± 0.013b	0.099 ± 0.038a	
Farmland watershed	0–100		0.117 ± 0.015a		0.088 ± 0.015b	0.118 ± 0.030a	0.092 ± 0.019b
	100–200		0.083 ± 0.010b		0.072 ± 0.010b	0.102 ± 0.036a	0.074 ± 0.009b
	200–300		0.087 ± 0.017b		0.079 ± 0.011b	0.112 ± 0.041a	0.085 ± 0.007b
	300–400		0.094 ± 0.014b		0.082 ± 0.015b	0.115 ± 0.039a	0.083 ± 0.010b
	400–500		0.088 ± 0.017b		0.080 ± 0.025b	0.113 ± 0.036a	0.085 ± 0.013b
	0–500		0.094 ± 0.021b		0.080 ± 0.012c	0.120 ± 0.017a	0.084 ± 0.014c

Different lowercase letters indicate significant differences among land use types within the same soil layer (*p* < 0.05). FE, native grassland (*Festuca elata*); RP, PT, and RX, vegetated restoration land (*Robinia pseudoacacia*, *Pinus tabuliformis*, and *Rosa xanthina*, respectively); ZM, farmland (*Zea mays*); MD, economic forestland (*Malus domestica*).

In the Artificial forest watershed, there was no significant difference in mean soil moisture content among RP, PT, and RX in the vegetated restoration land (*p* > 0.05), in the 0–200 cm soil layer, the mean soil moisture content was significantly higher in native grassland (FE) than in the vegetated restoration land (RP, PT, RX) and farmland (ZM) (*p* < 0.05), and in the 200–500 cm soil layer, the mean soil moisture content was significantly higher in both native grassland (FE) and farmland (ZM) than in the vegetated restoration land (RP, PT, RX) (*p* < 0.05). In the Artificial forest watershed, the average soil moisture content in the 0–500 cm soil layer of different vegetation types, in descending order, was farmland (ZM) (0.099 g/g), native grassland (FE) (0.091 g/g), vegetated restoration land (RX) (0.071 g/g), vegetated restoration land (PT) (0.070 g/g), and vegetated restoration land (RP) (0.068 g/g), with farmland (ZM) and native grassland (FE) all having significantly higher soil moisture content than vegetated restoration land (RP, PT, RX).

In contrast, in the 0–100 cm soil layer of Farmland watershed, the mean soil moisture content was significantly higher (*p* < 0.05) in farmland (ZM) and vegetated restoration land (RP) than in vegetated restoration land (RX) and economic forestland (MD), with no significant differences detected between economic forestland (MD)-vegetated restoration land (RP) pair and vegetated restoration land (RX)-economic forestland (MD) pair (*p* > 0.05), and in the 100–500 cm soil layer, the mean soil moisture content was significantly higher (*p* < 0.05) in farmland (ZM) than in vegetated restoration land (RP, RX), and economic forestland (MD). No significant differences have occurred in vegetated restoration land (RP), and statistical analysis revealed no detectable differences (*p* > 0.05) between the soil moisture of vegetated restoration land (RX) and economic forestland (MD). In the Farmland watershed, across the 0–500 cm profile, farmland (ZM) (0.120 g/g) maintained the highest average soil moisture content, followed in decreasing order by vegetated restoration land (RP) (0.094 g/g), economic forestland (MD) (0.084 g/g), and vegetated restoration land (RX) (0.080 g/g), in which the average soil moisture content of farmland (ZM) was significantly higher than vegetated restoration land (RP) (*p* < 0.05), the average soil moisture content of vegetated restoration land (RP) were significantly higher than vegetated restoration land (RX) and economic forestland (MD) (*p* < 0.05), and there was no significant difference between vegetated restoration land (RX) and economic forestland (MD) (*p* > 0.05).

### 3.3 Soil moisture depletion of different land use types in paired watersheds

**Figure 5:**
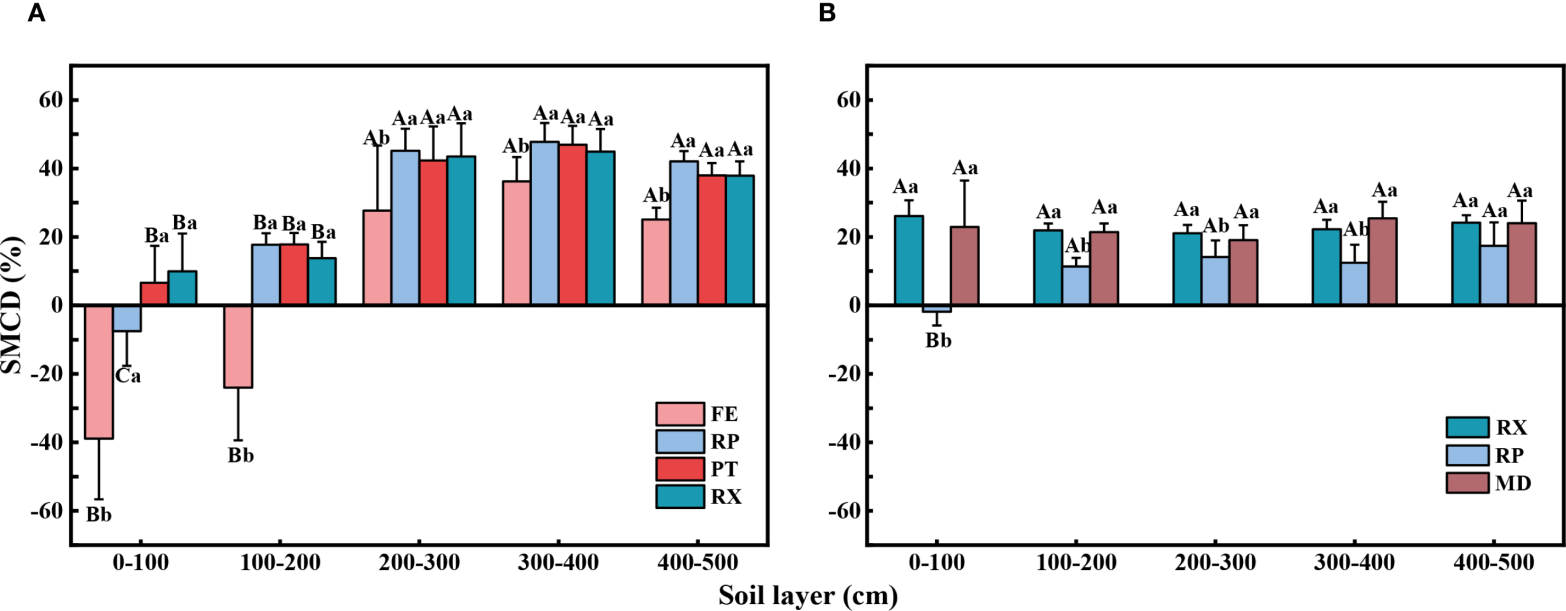
Variations in soil moisture depletion degree (SMCD) across soil layers under different land use types in two watersheds: **(A)** Artificial forest watershed and **(B)** Farmland watershed. Different lowercase and uppercase letters above the bars indicate significant differences between different land uses at the same soil depth and between the same land use at different soil depths (*p* < 0.05). FE, native grassland (*Festuca elata*); RP, PT, and RX, vegetated restoration land (*Robinia pseudoacacia*, *Pinus tabuliformis*, and *Rosa xanthina*, respectively); MD, economic forestland (*Malus domestica*).

Compared to farmland (ZM), both Artificial forest watershed and Farmland watershed exhibited varying degrees of soil moisture deficit (SMCD), with the deficit being more severe in Artificial forest watershed (**Figure 5**, **Table 4**).

**Table 4:** Soil moisture depletion degree (SMCD) of the 0–500 cm soil layers.

Land use	Soil depth (cm)	Native grassland	Vegetated restoration land			Economic forestland
		FE	RP	PT	RX	MD
Artificial forest watershed	0–500	5.22 ± 14.15b	29.03 ± 23.68a	30.32 ± 17.31a	30.00 ± 8.30a	
Farmland watershed	0–500		10.69 ± 7.38b		23.10 ± 2.01a	22.57 ± 2.46a

Different lowercase letters indicate significant differences among land use types within the same watershed (*p* < 0.05). FE, native grassland (*Festuca elata*); RP, PT, and RX, vegetated restoration land (*Robinia pseudoacacia*, *Pinus tabuliformis*, and *Rosa xanthina*, respectively); MD, economic forestland (*Malus domestica*).

Within the Artificial forest watershed, significant differences in SMCD were observed across soil layers for the same vegetation type. In native grassland (FE), the 0–100 cm and 100–200 cm layers showed no moisture deficit, while the 200–300 cm, 300–400 cm, and 400–500 cm layers exhibited deficits of 26.68%, 36.23%, and 25.10%, respectively. Similarly, no deficit was found in the 0–100 cm layer of vegetated restoration land (RP), but the 100–200 cm, 200–300 cm, 300–400 cm, and 400–500 cm layers showed SMCDs of 17.74%, 45.16%, 47.75%, and 42.03%, respectively. SMCD values were significantly higher in the 200–500 cm layers than in the 100–200 cm layer. For vegetated restoration land (PT and RX), all soil layers exhibited moisture deficits. In vegetated restoration land (PT), SMCD values reached 42.33% in the 200–300 cm, 300–400 cm, and 400–500 cm layers, which were significantly higher than the values in the 0–100 cm (6.60%) and 100–200 cm (17.79%) layers. Similarly, vegetated restoration land (RX) showed SMCDs of 43.48%, 44.92%, and 37.89% in the 200–300 cm, 300–400 cm, and 400–500 cm layers, all significantly higher than those in the 0–100 cm (9.95%) and 100–200 cm (13.77%) layers. Additionally, SMCD varied significantly among different vegetation types within the same soil layer. The vegetated restoration land (RP, PT, RX) exhibited significantly higher SMCD values than native grassland (FE) across all layers from 0–500 cm.

Within the Farmland watershed, the soil moisture content difference (SMCD) of vegetated restoration land (RX) and economic forestland (MD) did not differ significantly among soil layers. Vegetated restoration land (RP) did not experience water deficit in the 0–100 cm soil layer; however, soil moisture depletion was observed in the 100–200 cm, 200–300 cm, 300–400 cm, and 400–500 cm layers, with SMCDs of 11.35%, 14.11%, 12.48%, and 17.39%, respectively. In addition, significant differences in SMCD were observed among different vegetation types within the same soil layer, with vegetated restoration land (RX) and economic forestland (MD) exhibiting significantly higher SMCD values than vegetated restoration land (RP) across all soil layers from 0–500 cm.

### 3.4 Spatial distribution of soil moisture in different depths

**Figure 6:**
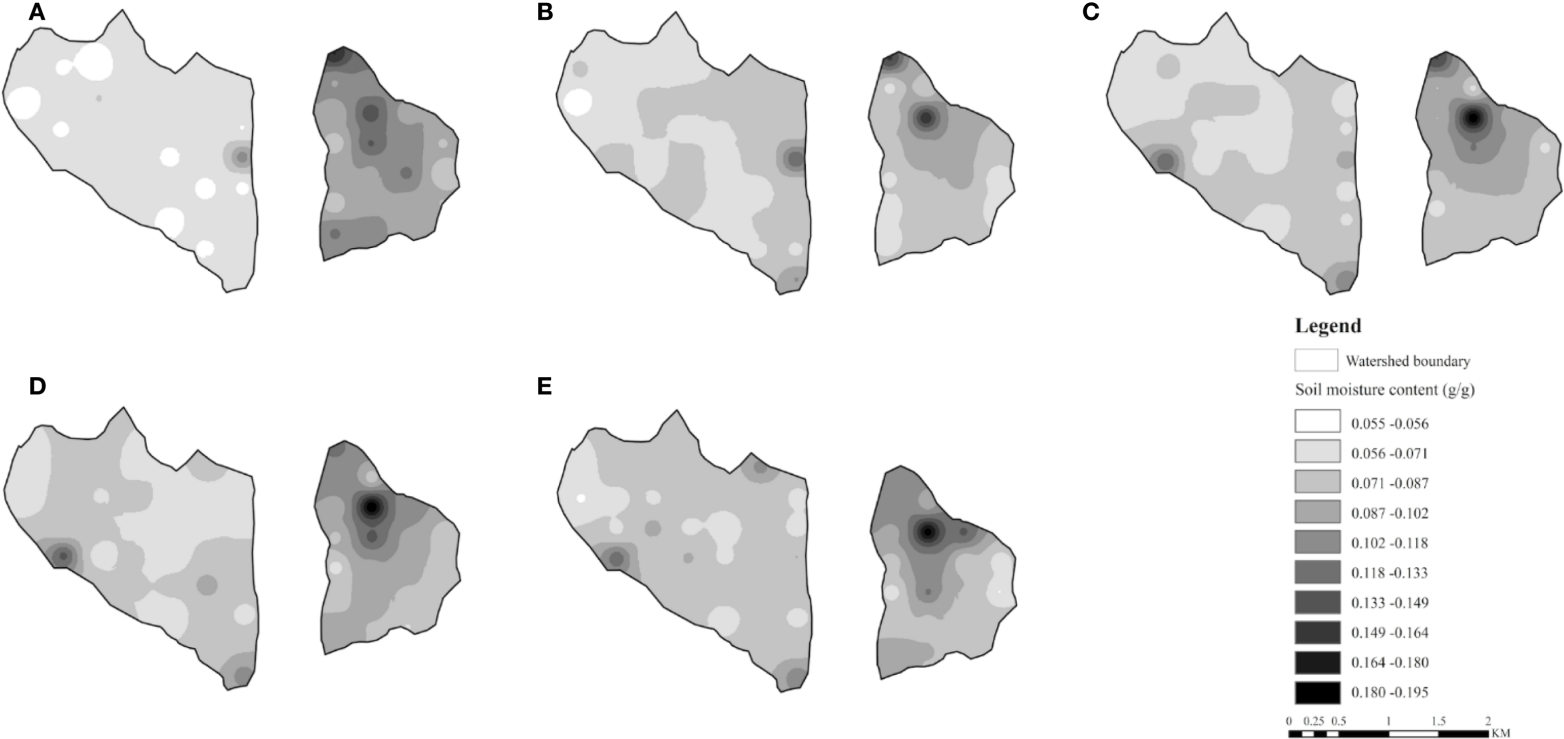
Soil moisture content interpolated by the IDW method in different soil depths. **(A)** 0–100 cm, **(B)** 100–200 cm, **(C)** 200–300 cm, **(D)** 300–400 cm, and **(E)** 400–500 cm.

**Figure 6** was generated with the Inverse Distance Weighting (IDW) method to characterize the spatial distribution of soil moisture in the Artificial forest watershed and the Farmland watershed. The results indicate that soil moisture distribution was closely associated with land use type, with relatively higher soil moisture in farmland (ZM) and native grassland (FE), and lower moisture levels at vegetated restoration land (RP, PT, RX). In the Farmland watershed, soil moisture showed a spatial pattern of high values in the northwest and low values in the southeast, while the Artificial forest watershed exhibited the opposite trend-lower values in the northwest and higher values in the southeast. The maximum mean soil moisture content in the 0–500 cm layer of the Artificial forest watershed occurred in native grassland (FE) (0.101 g/g at site 24), while the minimum occurred in vegetated restoration land (RP) (0.0526 g/g at site 6). In the Farmland watershed, the highest mean soil moisture was observed in farmland (ZM) (0.170 g/g at site 5), and the lowest in vegetated restoration land (RX) (0.067 g/g at site 14).

**Table 5** presents the statistics of soil moisture content across different soil layers in the two watersheds. In the 0–500 cm depth range, the coefficient of variation in the Artificial forest watershed ranges from 19.98% to 25.72%, with a standard deviation of 0.013–0.017. In the Farmland watershed, the coefficient of variation ranges from 29.95% to 31.29%, and the standard deviation ranges from 0.026 to 0.031. Both the coefficient of variation and standard deviation in the Farmland watershed are higher than those in the Artificial forest watershed.

**Table 5:** Basic statistics on soil moisture content (g/g) in two watersheds based on IDW.

Soil depth (cm)	Artificial forest watershed					Farmland watershed				
	Min g/g	Max g/g	Mean g/g	SD	CV (%)	Min g/g	Max g/g	Mean g/g	SD	CV (%)
0–100	0.043	0.109	0.061	0.013	25.72	0.073	0.164	0.105	0.031	29.95
100–200	0.045	0.132	0.073	0.016	21.74	0.061	0.161	0.085	0.026	30.66
200–300	0.057	0.131	0.074	0.015	20.71	0.066	0.191	0.092	0.029	30.99
300–400	0.056	0.136	0.074	0.016	21.41	0.065	0.192	0.096	0.028	29.55
400–500	0.054	0.126	0.078	0.016	19.98	0.055	0.185	0.094	0.029	31.29
0–500	0.053	0.101	0.070	0.017	24.29	0.067	0.171	0.096	0.030	31.25

SD, standard deviation; CV, coefficient of variation.

## 4 Discussion

### 4.1 Vertical distribution characteristics of soil moisture

The monitoring of deep soil moisture revealed that there were significant differences in soil moisture content between the paired watersheds (**Figure 2**). The Farmland watershed exhibited a markedly higher average soil moisture compared to the Artificial forest watershed. This phenomenon is consistent with the findings of Jia et al. (2017) and Liu et al. (2018), both of which indicated that, in typical vegetated restoration areas of the Loess Plateau, prolonged high-density afforestation has resulted in sustained depletion of deep soil moisture. The dynamics of soil moisture are directly influenced by precipitation, topography, and vegetation. Among these factors, precipitation constitutes a critical constraint on vegetated restoration and reconstruction in the Loess Plateau and represents one of the principal sources of soil moisture (Hou et al., 2018; Jin et al., 2018). In this study, the selected paired watersheds are adjacent to each other, with similar elevation and spatial precipitation distribution. Additionally, the Loess Plateau is characterized by deep soil layers, and soil moisture below the infiltration depth in restored vegetation areas remains relatively stable with minimal interannual fluctuations (Yang et al., 2012). Although the analysis in this study is based on monitoring data from a single growing season, short-term climatic variations have limited effects on deep soil moisture. Therefore, the primary factor responsible for the observed differences in deep soil moisture between the paired watersheds is likely the influence of vegetation roots under long-term restoration. The introduced vegetation in the Artificial forest watershed has developed extensive root systems, which, under identical rainfall conditions, exert a greater influence on soil moisture content compared to farmland and grassland (Wyatt et al., 2021). Moreover, the vegetation density in the Artificial forest watershed considerably exceeds the carrying capacity of natural precipitation, and rainfall is insufficient to meet the growth and development demands of such biomass-rich artificial plantations, thereby leading to excessive depletion of soil moisture (Jin et al., 2018). In contrast, the Farmland watershed is mainly cultivated with shallow-rooted annual crops such as ZM, which primarily consume surface soil moisture, allowing deeper moisture to be conserved.

By comparing soil moisture content across different soil depths in the two watersheds (**Figure 3**), it was found that the soil moisture content of the 0–100 cm layer in the Farmland watershed was 0.105 g/g, which was significantly higher than that in the Artificial forest watershed (0.061 g/g), representing a difference of 72.13%. In the 100–500 cm soil layer, however, the difference in soil moisture content narrowed to 16.44%, 24.32%, 29.73%, and 20.51%. This could be attributed to the fact that the lateral roots of the introduced vegetation species (RP, PT, RX) in the Artificial forest watershed are mainly distributed within the 0–100 cm layer, where considerable amounts of soil moisture are consumed during the growing season to sustain plant development (Cui et al., 2023; Zhou et al., 2022). Additionally, precipitation predominantly affects shallow soil moisture. Due to extensive vegetation cover in the Artificial forest watershed, canopy interception reduces surface infiltration, resulting in lower soil moisture content (O’Donnell et al., 2021; Wu et al., 2021). In the Farmland watershed, long-term cultivation practices have left crop residues and surface organic matter that help reduce bare soil exposure and minimize evaporation losses of surface soil moisture (Tuure et al., 2021). Furthermore, continuous tillage has altered the soil structure, promoted infiltration, and consequently increased soil moisture content in the 0–100 cm layer (Wang et al., 2017). The deep soil moisture content in the Artificial forest watershed remained lower than in the Farmland watershed. This may be because, under long-term vegetation restoration, plants tend to develop deeper roots to access moisture when the upper soil layers experience water stress, thereby depleting deep soil moisture and forming a dried soil layer (Tijdeman and Menzel, 2021; Wang et al., 2024).

In addition to root distribution and vegetation density, species-specific water use characteristics further elucidate the observed contrasts in deep soil moisture. Sap flow and eddy covariance studies in the Loess Plateau have shown that R*obinia pseudoacacia* (RP) exhibits annual evapotranspiration levels of approximately 480–550 mm·yr^−1^. Due to its deep root system and high canopy transpiration demand, the overall water consumption of RP often exceeds the mean annual precipitation (Wu et al., 2021). By contrast, Zea mays (ZM) generally has an annual evapotranspiration of 350–450 mm·yr^−1^, which is more closely aligned with regional precipitation inputs (Liu et al., 2010). This indicates that ZM primarily relies on seasonal rainfall, whereas RP continuously depletes deep soil moisture. These interspecific differences in water use efficiency further demonstrate the importance of considering evapotranspiration demand when selecting restoration species in water-limited regions such as the Loess Plateau.

### 4.2 Mechanisms driving the spatial variability of deep soil moisture

This study found that the spatial heterogeneity of soil moisture in the Farmland watershed was considerably higher than that in the Artificial forest watershed (**Figure 6**). Across the 0–500 cm profile, the coefficient of variation in the Farmland watershed reached 31.25%, exceeding the 24.29% observed in the Artificial forest watershed. In all soil layers, the coefficient of variation in the Farmland watershed was consistently higher, which aligns with the findings of Yang et al. (2014), who reported that the distribution of soil moisture in the Farmland watersheds is more complex compared to the Artificial forest watersheds. The primary reason for this difference lies in the contrasting land use structures between the two watersheds. The Artificial forest watershed has undergone long-term vegetation restoration, resulting in a predominantly monocultural vegetation pattern dominated by artificial forest, which leads to relatively uniform soil moisture distribution. By contrast, the Farmland watershed comprises a mosaic of farmland, economic forest, and shrubland, thereby increasing the spatial variability of soil moisture distribution. The significant differences in deep soil moisture between the two watersheds indicate that complex land use structures are associated with higher spatial heterogeneity of deep soil moisture, whereas large-scale vegetation restoration tends to enhance the spatial homogeneity of deep soil moisture.

In addition, soil and water conservation projects also play a crucial role in shaping the spatial pattern of deep soil moisture. Among the 22 experimental sites in the Farmland watershed, sites 1, 5, 8, and 13 exhibited substantially higher soil moisture than other sites. This is likely because these sites were located within check dams, which are typical soil and water conservation measures in the Farmland watersheds. Check dams promote water infiltration, reduce surface runoff, and control soil erosion, thereby contributing to increased soil moisture (Helman and Mussery, 2020; Luan et al., 2022).In the Artificial forest watershed, the highest soil moisture in the 0–200 cm layer was observed at site 24, possibly because this site was situated in native grassland (FE). FE has shallow root systems, resulting in less uptake of deep soil moisture (Ye et al., 2019). Additionally, the canopy height and biomass of FE are relatively low, leading to lower canopy interception and evaporation compared to other vegetation types. As a result, a greater proportion of precipitation infiltrates into the soil, maintaining higher soil moisture content (Jian et al., 2015). In the 200–500 cm layer, the highest soil moisture was recorded at sites 19 and 34. This may be attributed to site 19 being located in farmland (ZM), where tillage practices modify the structure of surface soil aggregates, increase soil porosity, and facilitate infiltration of rainfall, thereby accelerating the percolation of moisture into deeper soil layers. Site 34 was located at the outlet of the watershed, in a relatively low-lying terrain position. Under identical precipitation or snowmelt input conditions, water tends to accumulate along the slope and infiltrate into deep soil layers, forming a moisture convergence zone and resulting in higher deep soil moisture content (Chang et al., 2024).

### 4.3 Implications for vegetation restoration

In the Loess Plateau region, deep soil moisture plays a critical role in sustaining the growth and ecological functions of artificial vegetation (**Figure 4**). This study demonstrated that, in the Artificial forest watershed, soil moisture content across all soil layers under vegetated restoration land (RP, PT, RX) was significantly lower compared to farmland (ZM) and native grassland (FE). Specifically, the average soil moisture content (0–500 cm) in the Artificial forest watershed was significantly higher in ZM (0.099 g/g) and FE (0.091 g/g) than in RP (0.068 g/g), PT (0.070 g/g), and RX (0.071 g/g). A similar pattern was observed in the Farmland watershed, where the difference in moisture content between vegetated restoration land and farmland was also significant. In the Farmland watershed, farmland (ZM) showed the highest average moisture content in the 0–500 cm soil layer (0.120 g/g), significantly higher than RP (0.094 g/g), MD (0.080 g/g), and RX (0.080 g/g). This difference may be attributed to the annual crop (ZM), which has a shallow root system and short life cycle, consuming mainly surface soil moisture and thus conserving deeper soil moisture (Shen et al., 2014; Xiao et al., 2014). Native grassland (FE), despite having year-round coverage, also possesses relatively shallow roots that consume less deep-layer moisture, thus maintaining higher deep soil moisture levels (Zhang et al., 2024). In contrast, vegetated restoration sites (RP, PT, RX) have deeper root systems and, over prolonged restoration periods, continuously consume deep soil moisture through transpiration, resulting in a lower soil moisture content (Liu et al., 2024a).

The difference in soil moisture content between vegetated restoration sites and farmland can represent the extent of soil moisture depletion caused by vegetation restoration. Results from this study (**Figure 5**) indicate that the soil moisture deficit at vegetated restoration sites (RP, PT, and RX) was significantly greater than that of native grassland (FE) within the 0–500 cm soil layer in the Artificial forest watershed. Specifically, in terms of soil depth, the soil moisture content deficit (SMCD) for RP, PT, and RX in the 0–100 cm soil layer was −7.52%, 6.60%, and 9.90%, respectively, while the most severe water depletion occurred in the 300–400 cm soil layer, with deficits of 47.75%, 46.94%, and 44.90%, respectively. This pattern demonstrates that artificial vegetation significantly depleted deep soil moisture reserves, with a more pronounced depletion effect in deeper soil layers, leading to the formation of a deep, dry soil layer that gradually restricts forest growth (Zhang et al., 2018). In contrast, soil moisture depletion by artificial vegetation in the Farmland watershed did not reach such extreme levels. In the Farmland watershed, RP did not exhibit a soil moisture deficit in the 0–100 cm layer, and deeper layers showed only moderate deficits ranging from 11.35% to 17.39%. This moderate deficit may result from the smaller scale and lower density of the RP in the Farmland watershed compared to the Artificial forest watershed, resulting in relatively lower deep soil moisture consumption (Liu et al., 2024b; Zhang et al., 2018). These findings suggest that large-scale vegetation restoration, particularly with deep-rooted species, significantly reduces deep soil moisture content, and that the depletion mechanism is closely related to the water-use characteristics of vegetation. This interpretation is consistent with Zhou et al. (2024), who emphasized that vegetation type exerts a strong influence on deep soil moisture dynamics, with deep-rooted broadleaf species contributing more to deep soil drying, whereas shallow-rooted vegetation maintains a more balanced soil moisture regime. The scarcity of deep soil moisture therefore poses a critical challenge to the sustainability of afforestation and vegetation restoration efforts in water-limited regions. Consequently, it is advisable to avoid large-scale afforestation with deep-rooted monocultures, as this practice may expedite the development of deep dry soil layers. The density of afforestation efforts should be optimized based on rigorous scientific analysis of local hydrological conditions. For existing plantations, adaptive management strategies—such as thinning to decrease stand density or replacing species—should be implemented to mitigate deep soil moisture deficits and promote the long-term sustainability of vegetation restoration efforts.

## 5 Conclusions

This study has systematically analyzed the vertical distribution characteristics and spatial variability of soil moisture (0–500 cm) in typical paired watersheds (Farmland watershed and Artificial forest watershed) within the Caijiachuan watershed in the hilly-gully region of the Loess Plateau in western Shanxi Province. The results demonstrated that long-term vegetation restoration substantially reduced deep soil moisture reserves. The mean soil moisture content in the Artificial forest watershed (0.070 g/g) was significantly lower than that in the Farmland watershed (0.096 g/g), indicating that artificially introduced deep-rooted vegetation markedly intensified soil moisture deficits in the 200–500 cm layers. Farmland and native grassland exhibited stronger capacities for maintaining deep soil moisture, whereas vegetation restoration sites consistently showed pronounced deep moisture depletion. In addition, the spatial heterogeneity of deep soil moisture was significantly higher in the Farmland watershed, while long-term afforestation promoted a more homogeneous spatial distribution of soil moisture content. Overall, the findings indicate that large-scale vegetation restoration dominated by deep-rooted species can lead to substantial deep soil desiccation, posing potential risks to vegetation sustainability and regional water balance. Therefore, future land-use planning and vegetation restoration efforts in the Loess hilly-gully watershed should carefully balance the trade-offs between ecological restoration and soil moisture availability, scientifically select and arrange appropriate vegetation types, and implement adaptive management strategies to ensure the long-term sustainability of ecosystem functions.

## Data Availability

The raw data supporting the conclusions of this article will be made available by the authors, without undue reservation.
